# Assessing the off-target movement of tebufenozide in forested ecosystems: implications for vernal pond ecosystems

**DOI:** 10.1007/s10661-025-14908-4

**Published:** 2026-01-03

**Authors:** Mason S. Ward, Hlengilizwe Nyoni, Odette Mina, Jon N. Sweetman

**Affiliations:** 1 Department of Ecosystem Science and Management, The Pennsylvania State University, University Park, PA USA; 2 Energy and Environmental Sustainability Laboratories, Institute for Energy and the Environment, The Pennsylvania State University, University Park, PA USA

**Keywords:** Pesticide drift, Aquatic contamination, Forest ecosystem dynamics, Amphibian habitats, Wetlands

## Abstract

The widespread use of pesticides has significantly contributed to managing pest populations in both agricultural and forest ecosystems, yet concerns about their unintended impacts on non-target habitats continue to grow. Tebufenozide, a pesticide commonly used to control forest defoliator pests, including spongy moth (*Lymantria dispar dispar*), is known for its selective action on Lepidopteran larvae. Despite its targeted mode of action, the potential transport and fate of tebufenozide into sensitive forested aquatic habitats, such as vernal ponds, is not well understood. This study examines the spatial distribution of tebufenozide in 41 vernal ponds located within three state forests in central Pennsylvania (Bald Eagle, Rothrock, and Tuscarora) by analyzing sediment and water samples collected within and outside designated spray blocks. Tebufenozide was detected in 39 water samples and 40 sediment samples, including 27 unsprayed water and sediment samples, indicating possible pesticide drift or runoff into non-target areas. We used a Mann-Whitney *U* test to reveal significantly higher concentrations of tebufenozide in ponds within spray blocks for both sediment (*W* = 241.5, *p* = 0.0161) and water (*W* = 316.5, *p* = 2.769e-06). Tebufenozide concentrations were higher in ponds closer to spray zones, suggesting proximity influences pesticide levels, though no clear directional dispersal patterns emerged. These findings underscore the vulnerability of vernal ponds, essential breeding habitats for amphibians and other organisms, to pesticide contamination. Enhanced management strategies, such as wider buffer zones and alternative pest control measures, may be necessary to safeguard these critical ecosystems.

**Supplementary Information:**

The online version contains supplementary material available at 10.1007/s10661-025-14908-4.

## Introduction

Pesticides are integral to the global management of agricultural and forest ecosystems, providing a critical tool for controlling pest populations that threaten crops, forests, and overall ecological balance. Their widespread use has been pivotal in preventing large-scale damage from invasive species and pests, such as the spongy moth (*Lymantria dispar dispar*), which has caused extensive defoliation and has undermined forest resilience across the Northeastern United States (Leroy et al., 2023). However, the environmental impact of pesticides, especially in non-target ecosystems, remains a growing concern (Malhotra et al., 2021). As pesticides move beyond their intended application areas, they can unintentionally affect surrounding habitats and ecosystems, posing risks to biodiversity and ecological integrity.

Among the various pesticides used in forest management, tebufenozide is valued for its specificity in targeting the molting processes of Lepidopteran larvae, minimizing acute toxicity to non-target species (Dhadialla & Jansson, 1999). However, despite its selective mode of action, research on tebufenozide’s impact on non-target aquatic organisms and its environmental behavior remains limited (Leroy et al., 2023; Sundaram et al., 1996), although there is some evidence of potential impacts to non-target freshwater invertebrates (Song et al., 1997; Tassou & Schulz, 2013). Vernal ponds are unique, ephemeral aquatic ecosystems that provide critical habitats for a wide range of organisms, particularly amphibians and invertebrates. These ponds, which fill seasonally and dry up in the summer, support breeding populations of amphibians such as wood frogs (*Lithobates sylvaticus*) and spotted salamanders (*Ambystoma maculatum*), as well as a variety of aquatic insects and microorganisms that form essential components of forest food webs (Colburn, 2004; Colburn et al., 2008). Temporary water bodies, such as vernal ponds, can be vulnerable to chemical contamination due to their isolation from larger water bodies, unique hydrological cycles, and lack of protection by no-spray buffer zone requirements (Mann et al., 2003; Thompson et al., 2004). Their limited capacity for dilution and potential for contaminant accumulation make vernal ponds particularly susceptible to pesticide exposure (Battaglin et al., 2009; Hayden et al., 2022). Even low concentrations of pesticides in these habitats have the potential to disrupt critical ecological processes. For example, atrazine exposure as low as 0.1 ppb has been shown to induce hermaphroditism and disrupt endocrine function in amphibians (Hayes et al., 2002). Similarly, experimental studies have demonstrated that herbicides and insecticides can alter amphibian survival and biomass by both direct toxicity and indirect effects mediated through predator-prey interactions (Relyea et al., 2005). These findings highlight the vulnerability of vernal pond organisms to pesticide exposure, even at low or environmentally realistic concentrations.

Pesticide drift and runoff have been extensively studied in agricultural settings. The simplified, open landscapes make such processes feasible to model and manage (Tudi et al., 2021). Agricultural fields are subject to well-documented mechanisms of pesticide dispersal, including spray drift (Maybank et al., 1978) and precipitation runoff (Jergentz et al., 2005; Willis & McDowell, 1982), which can transport chemicals far beyond their intended zones. The behavior of pesticides in forested environments remains largely underexplored, in part due to the complex terrain, dense vegetation, and variable topography that introduce additional challenges that can complicate containment and predictability of pesticide movement (Neary et al., 1993; Payne, 2000). Additionally, canopy cover in forests plays a crucial role, modifying microclimates by altering air flow, humidity, and rainfall interception, which influence pesticide deposition (Payne, 1998; Wallace et al., 1995). Environmental factors like soil properties, hydrology, and local weather patterns have been shown to also impact pesticide mobility and persistence (Froger et al., 2023; Neary et al., 1993; Payne, 2000). In Pennsylvania, tebufenozide is commonly used to manage spongy moth (*Lymantria dispar dispar*) populations on state lands. This chemical control method is often preferred over alternatives like *Bacillus thuringiensis* var. *kurstaki* (*Btk*) for severe infestations (PA DCNR - Bureau of Forestry, 2023). The Pennsylvania Department of Conservation and Natural Resources (DCNR) enforces guidelines to mitigate environmental impact, including requirements for site-specific environmental reviews and adherence to buffer zones near open water bodies and sensitive habitats. Despite these measures, Pennsylvania’s diverse topography and dense vegetation can facilitate unintended pesticide movement into vernal ponds, raising concerns about potential accumulation in aquatic ecosystems that may lack adequate protection.

This study aims to examine the spatial distribution of tebufenozide in vernal ponds across three state forests in central Pennsylvania, USA. By analyzing water and sediment samples collected both within and outside designated spray blocks, we seek to assess the extent of tebufenozide’s dispersion beyond its intended application zones. We hypothesize that tebufenozide concentrations will be higher in ponds within spray blocks, with detectable levels found in ponds outside these areas indicative of unintentional spread. This research aims to improve our understanding of pesticide dynamics in forested environments and provide insights to guide management practices that protect the ecological integrity of vernal ponds.

## Materials and methods

### Study sites and sampling

**Fig. 1:**
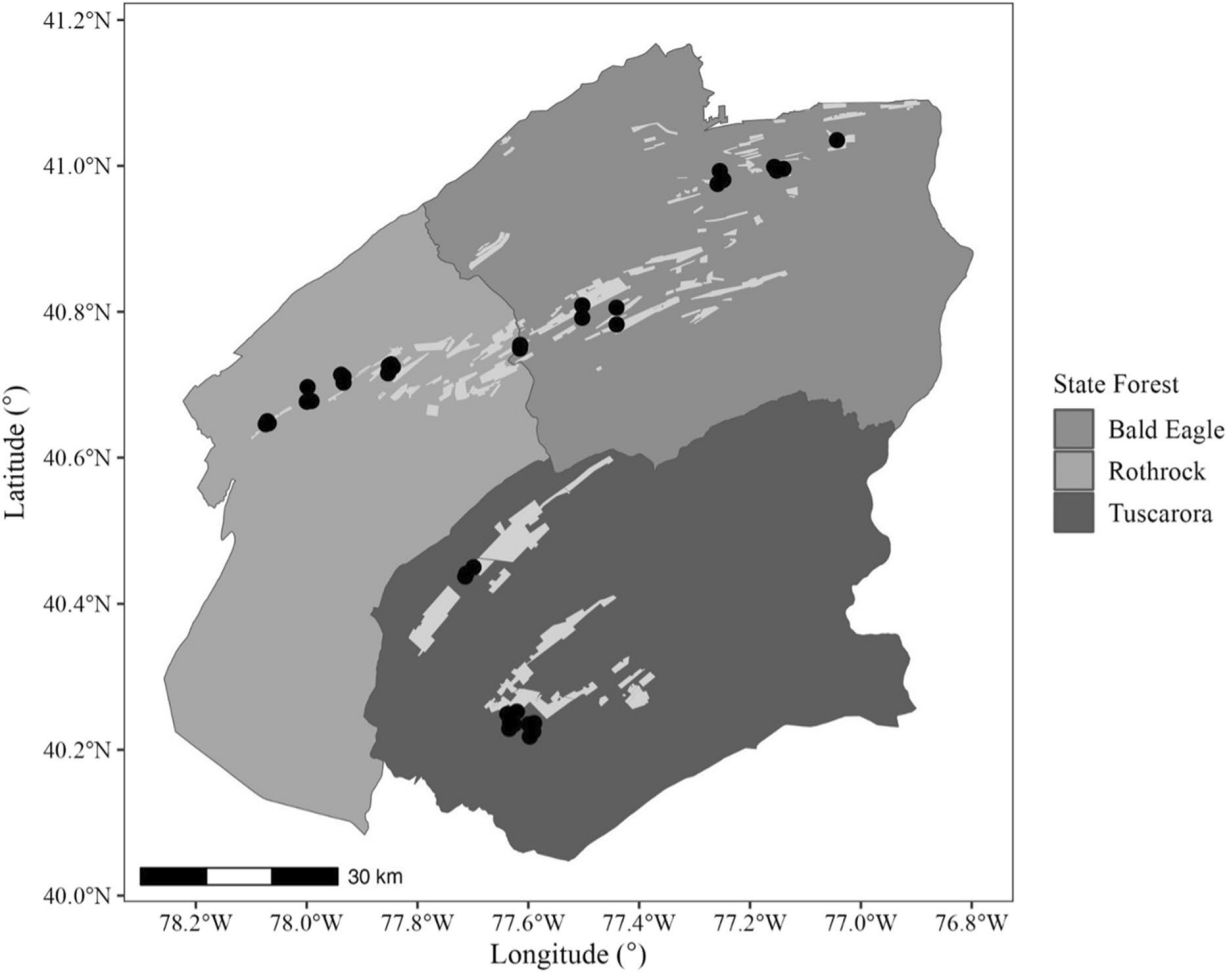
A map illustrating the locations of the 41 vernal ponds sampled within three state forests in Pennsylvania: Bald Eagle, Rothrock, and Tuscarora. Lightly shaded areas represent spray blocks targeted during spongy moth (*Lymantria dispar dispar*) suppression programs over the past 3 years. Black circles indicate pond sites

A total of 41 ponds were sampled across three state forests in Pennsylvania: Bald Eagle (*n* = 15), Rothrock (*n* = 13), and Tuscarora (*n* = 13; Fig. 1). Of these ponds, 13 were located within designated spray blocks, while 28 were situated outside these areas. Spray block designations were obtained from Pennsylvania DCNR records, which maintain a 3-year rotation for tebufenozide applications where transects are only sprayed once during this cycle. Pond sites in this study were randomly selected prior to the release of spray block data and therefore were not chosen based on treatment history. After sampling was completed, ponds were classified as “sprayed” or “unsprayed” according to their location relative to spray block boundaries.

Sampling was conducted throughout the month of May 2024, coinciding with the peak period of pesticide application, to capture potential tebufenozide presence and accumulation in pond water and sediment. At each of the 41 ponds, paired samples of water and sediment were collected for pesticide analysis, resulting in a total of 82 samples. Sediment samples were extruded using a 68-mm-diameter sediment corer (Aquatic Research Instruments Universal Gravity Corer), with the top 5 cm designated for analysis to focus on recent deposition. Vegetation and surface leaf litter were removed prior to sampling to ensure only bare sediment was analyzed. Water samples were collected from approximately 10–15 cm below the surface near the center of each pond using 250-mL amber glass VWR® TraceClean® Wide Mouth Packers, which are certified to EPA cleaning procedures (power directive 9240.0–95a), to minimize contamination and capture representative water column conditions. All sediment and water samples were collected and stored in 250-mL amber glass VWR® TraceClean® Wide Mouth Packers. Sediment samples were frozen immediately upon collection to preserve their integrity, while water samples were maintained at 4 °C and shipped to The Pennsylvania State University (PSU) Environmental Contaminants Analytical Laboratory (ECAL). Pond characteristics and detailed analytical methodology are provided in the Supplementary Material.

### Statistical analysis

Prior to statistical analyses, one water sample was removed due to being below the limit of quantification (LOQ). All statistical analyses were performed using R version 4.2.3. Tebufenozide concentrations in water and sediment samples were compared between ponds inside and outside spray blocks using the non-parametric Mann-Whitney *U* test to assess differences in pesticide accumulation (R Core Team, 2025). This test was chosen due to the non-normal distribution of the concentration data. To examine potential spatial gradients of pesticide contamination, a linear regression was conducted to assess the relationship between tebufenozide concentrations in ponds outside of designated spray blocks and their proximity to the nearest spray block. Spatial distribution patterns of tebufenozide concentrations were visualized using spatial mapping techniques to examine potential dispersal trends in ggplot (Wickham, 2016). All statistical tests were conducted at a significance level of *α* = 0.05.

## Results and discussion

**Fig. 2:**
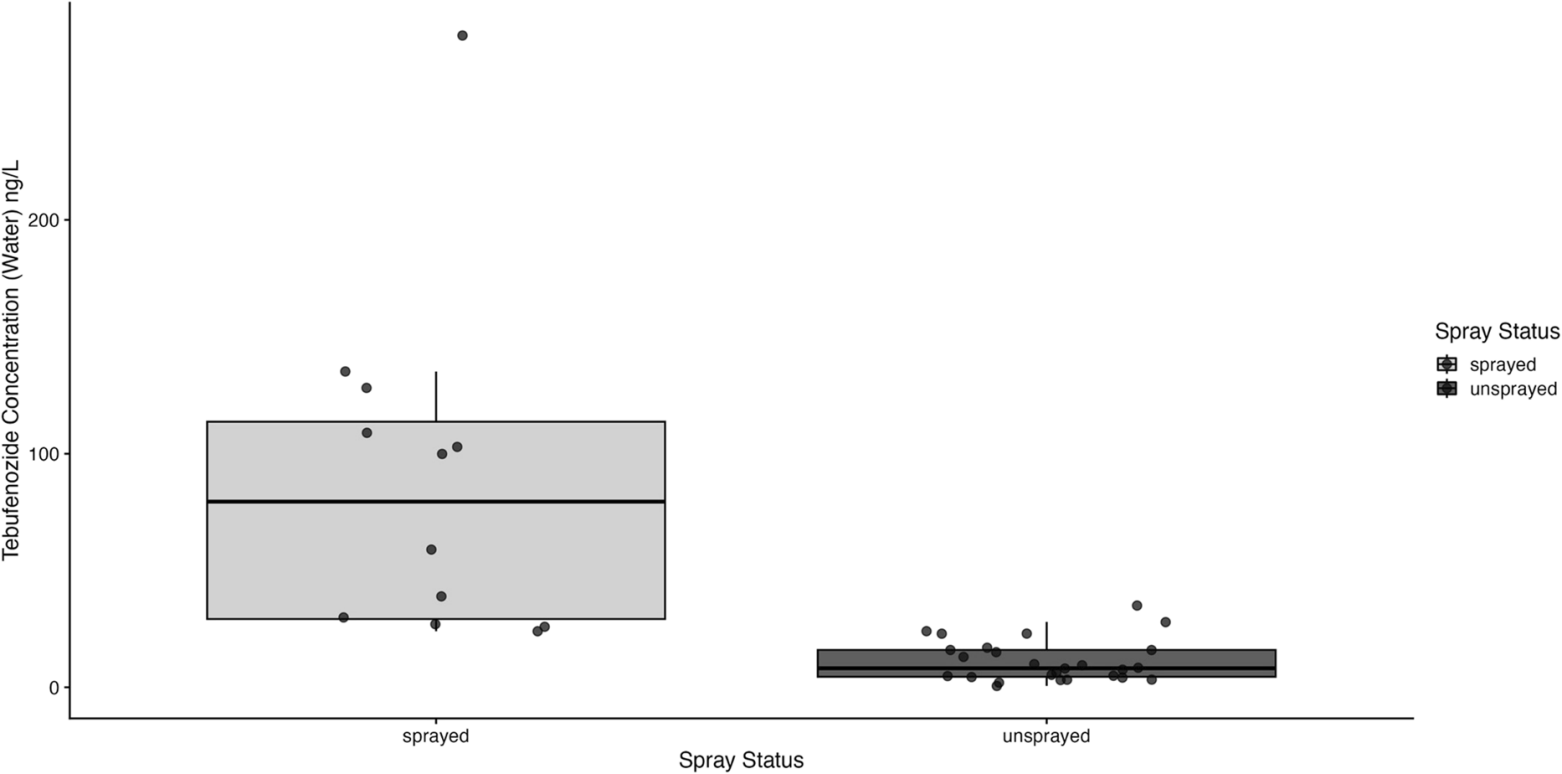
Box plot with jittered points showing tebufenozide concentrations in water samples collected from vernal ponds located within and outside of designated spray blocks. Jittered points represent individual site measurements. Concentrations were significantly higher in ponds located within sprayed areas

**Fig. 3:**
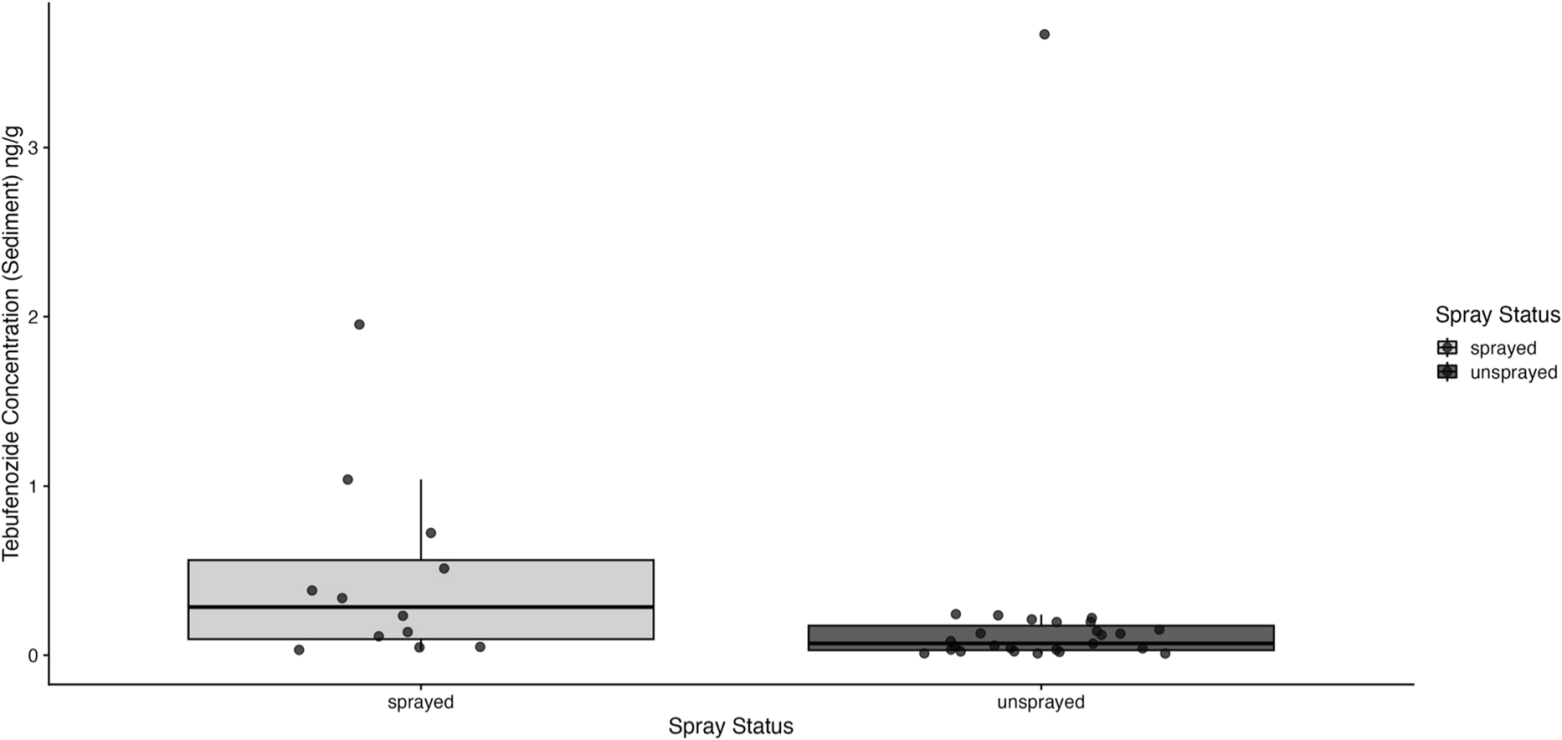
Box plot with jittered points showing tebufenozide concentrations in sediment samples collected from vernal ponds located within and outside of designated spray blocks. Jittered points represent individual site measurements. Concentrations were significantly higher in ponds located within sprayed areas

Tebufenozide was detected in 39 of the water samples and 40 of the sediment samples collected across the 41 vernal ponds. Detection was nearly universal in unsprayed areas, with only one pond lacking measurable levels, resulting in 27 of 28 water and sediment samples containing tebufenozide. In contrast, tebufenozide was found in 12 of 13 water samples and all 13 sediment samples within sprayed areas. One water sample in an unsprayed area had a concentration below the limit of quantification (LOQ). Tebufenozide concentrations were significantly elevated in sprayed areas for water samples (*W* = 304.5, *p*-value = 3.335e-06) and in sediment samples (*W* = 241.5, *p*-value = 0.0161; Figs. 2 and 3).

**Fig. 4:**
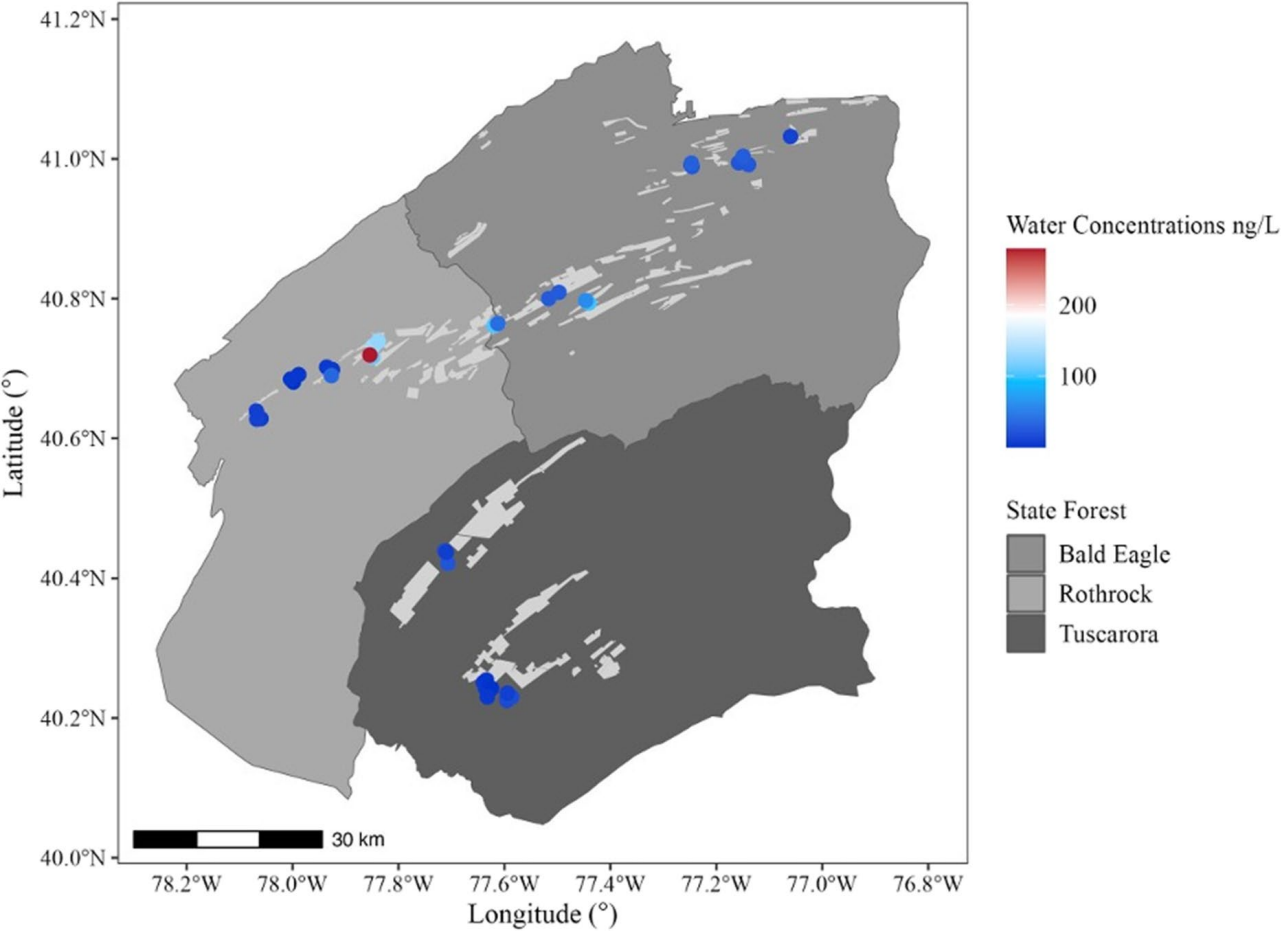
A spatial map illustrating tebufenozide concentrations in water samples across the 41 vernal ponds. Lightly shaded areas represent spray blocks targeted during spongy moth (*Lymantria dispar dispar*) suppression programs over the past 3 years. Concentration levels are visualized using a gradient scale, with higher levels generally occurring closer to spray blocks

**Fig. 5:**
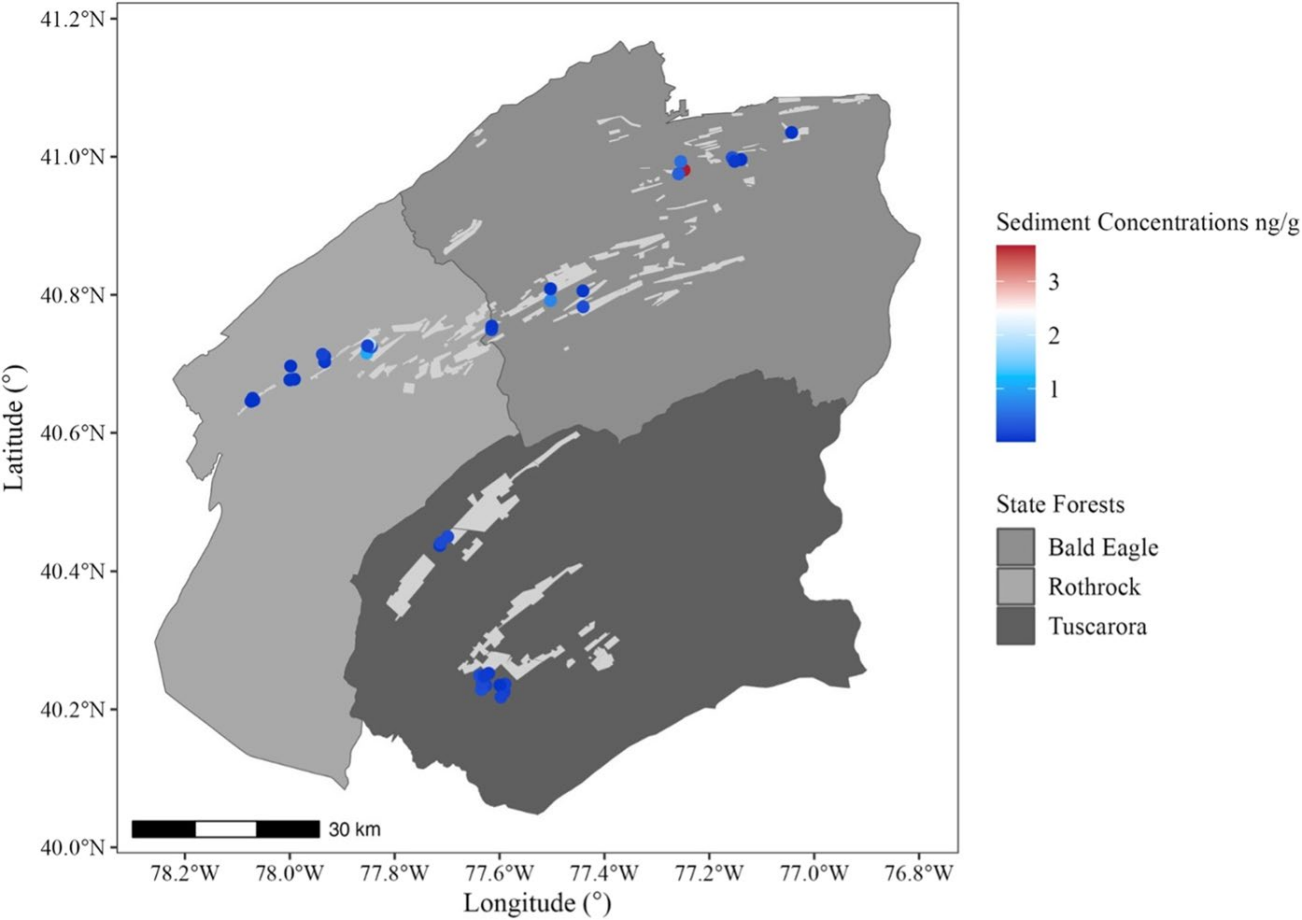
A spatial map showing tebufenozide concentrations in sediment samples across the 41 vernal ponds. As with the water samples, a gradient scale represents concentration levels, with higher levels detected in ponds nearer to spray blocks

**Fig. 6:**
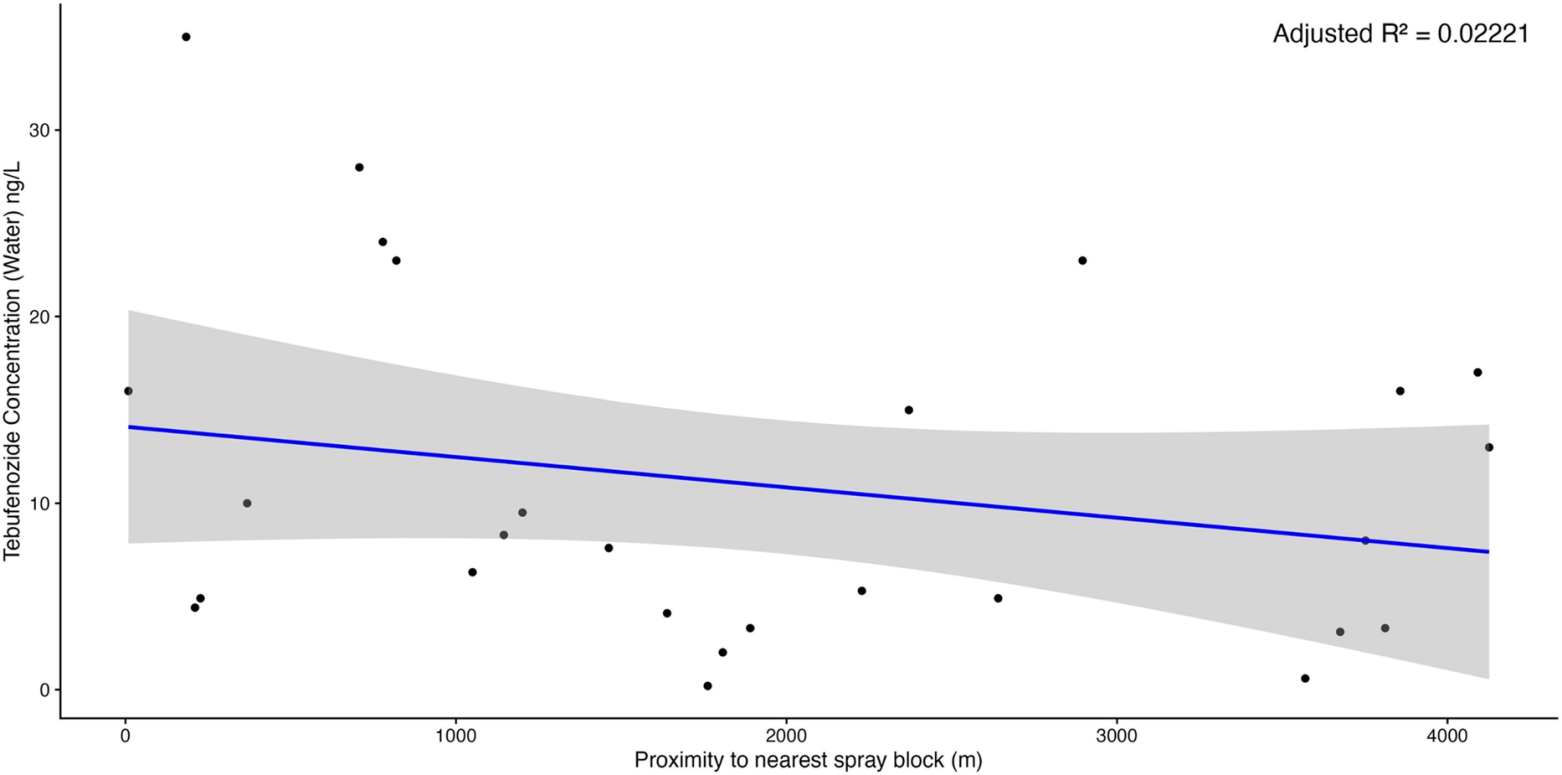
Linear regression showing the relationship between distance from spray blocks and tebufenozide concentrations in water. Shaded areas represent 95% confidence intervals around the fitted regression lines

**Fig. 7:**
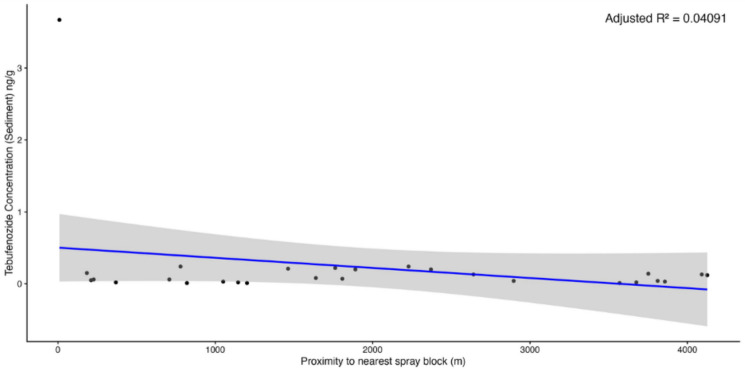
Linear regression showing the relationship between distance from spray blocks and tebufenozide concentrations in sediment. Shaded areas represent 95% confidence intervals around the fitted regression lines

When mapping tebufenozide concentrations across the study area to explore potential spatial trends, no consistent directional pattern emerged. Patterns emerged suggesting that ponds located closer to spray blocks displayed elevated concentrations of tebufenozide (Figs. 4 and 5), though linear regressions indicated weak, non-significant negative relationships between distance from spray blocks and tebufenozide concentrations in both water (*F*(1, 25) = 1.59, *p*-value = 0.2189, adjusted *R*^2^ = 0.02221; Fig. 6) and sediment samples (*F*(1, 25) = 2.11, *p*-value = 0.1589, adjusted *R*^2^ = 0.04091; Fig. 7). These results suggest a potential trend of declining concentration with increasing distance, although the relationships were not statistically significant. Overall, these findings indicate that tebufenozide disperses into non-target areas and that proximity to spray zones may influence pesticide levels in nearby ponds.

## Discussion

This study focused on the spatial distribution of tebufenozide in vernal ponds across three state forests in Pennsylvania, underlining the potential complex movement of pesticide dispersal in forested environments. Our findings reveal that tebufenozide, commonly used for spongy moth suppression, is not confined to designated spray areas, but was present in almost all vernal ponds sampled in the area, in both water and sediment. Its presence in unsprayed vernal ponds points to potential off-target movement, which raises ecological concerns for these sensitive aquatic habitats.

### Tebufenozide dispersal patterns and influencing factors

Our analysis revealed significantly higher tebufenozide concentrations in ponds within spray blocks compared to those outside of these areas. Tebufenozide concentrations in sediment and water were consistently elevated in ponds located within treated areas, underscoring the direct impact of targeted pesticide application. The results of the Mann-Whitney *U* tests confirm that pesticide application within designated areas contributes to increased contamination in both sediment and water samples. Nonetheless, the detection of tebufenozide in numerous unsprayed ponds suggests that dispersal mechanisms such as wind drift, precipitation-driven runoff, and soil leaching may facilitate the unintended spread of the pesticide (Neary et al., 1993). While these dispersal processes are well-documented in agricultural landscapes (Tudi et al., 2021), they remain underexplored in forested systems, where topographical variation, dense vegetation, and intricate hydrology can exacerbate containment challenges (Neary et al., 1993). Moreover, the presence of tebufenozide in ponds located in unsprayed areas raises questions about the long-range transport potential of pesticides in forested landscapes, a topic that requires further investigation.

The spatial mapping did not reveal a consistent directional pattern of tebufenozide dispersal, suggesting that movement in forested landscapes is more erratic and influenced by site-specific environmental features. Site-specific factors, like localized wind patterns and variations in vegetation density, could lead to a highly heterogeneous distribution of pesticide residues. However, higher pesticide concentrations were generally observed in ponds closer to spray blocks, indicating that proximity remains a significant factor in exposure risk. This observation is supported by weak negative relationships between distance from spray blocks and tebufenozide concentrations in sediment and water, though these trends were not statistically significant. For sediment, the apparent relationship was largely influenced by a single high concentration from a pond located near a spray zone. The low power of the models suggests that other environmental factors, such as wind, canopy cover, and hydrology, may also shape off-target contamination. These observations are consistent with prior research highlighting the roles of forest structure and canopy cover in influencing pesticide drift and deposition (Payne, 2000).

### Ecological implications for vernal ponds

The detection of tebufenozide in vernal ponds, even at low concentrations, raises significant ecological concerns. Vernal ponds are crucial habitats for amphibians, macroinvertebrates, and microbial communities, which are sensitive to chemical disturbances (Colburn, 2004). These isolated water bodies have a limited capacity for dilution, making them vulnerable to chemical accumulation (Hayden et al., 2022). Low-level exposure to specific pesticides has been linked to developmental abnormalities in amphibians (Campbell Grant et al. 2020), including delayed metamorphosis and impaired immune function. For example, Greulich and Pflugmacher (2003) reported delayed metamorphosis associated with the pyrethroid insecticide cypermethrin, while Christin et al. (2004) found impaired immune responses following exposure to a mixture of atrazine, metribuzin, endosulfan, lindane, aldicarb, and dieldrin. Previous studies have also demonstrated that contamination with specific compounds can impair amphibian development, reduce invertebrate populations, and disrupt microbial processes critical to forest food webs. For instance, atrazine (Hayes et al., 2002), malathion and glyphosate-based herbicides (Relyea et al., 2005), and the pesticide mixtures reported by Christin et al. (2004) all negatively affect amphibian and invertebrate communities. These disruptions may further cascade through ecosystems, altering predator-prey interactions and nutrient cycling in forested wetlands (Battaglin et al., 2009; Chagnon et al., 2015). Our findings underscore the need for more comprehensive risk assessments that account for the unique vulnerabilities of vernal ponds and the broader ecological impacts of pesticide drift.

## Conclusions

Despite existing mitigation strategies, such as buffer zones around larger waterbodies, our results suggest that current guidelines may be insufficient to prevent off-target contamination. To better protect vulnerable habitats, forest management practices should consider enhancing buffer zone widths, incorporating more detailed environmental assessments that account for the complex hydrology and wind patterns of forested landscapes, and exploring alternative pest management strategies such as *Bacillus thuringiensis kurstaki* (*Btk*) in areas near sensitive aquatic ecosystems. Wider buffer zones have been proposed as a possible solution, but their effectiveness in forested landscapes remains largely untested. Future research should include long-term monitoring to understand the persistence and cumulative effects of tebufenozide in vernal ponds. Monitoring efforts would also benefit from including other currently approved forest insecticides and historically applied compounds that may continue to influence aquatic ecosystems. Controlled experiments that simulate forest conditions to further explore the pathways driving pesticide dispersal. These studies should prioritize understanding how seasonal variations and extreme weather events influence pesticide movement and persistence. By deepening our understanding of these dynamics, we can develop more effective management practices that balance pest control with the preservation of the ecological integrity of forested aquatic habitats.

## Supplementary Information

Below is the link to the electronic supplementary material.

ESM 1 Supplementary Material 1 (DOCX 263 KB)

## Data Availability

The dataset supporting the findings of this study is publicly available: (10.26207/g55c-bj36).

## Declarations

“All authors have read, understood, and have complied as applicable with the statement on “Ethical responsibilities of Authors” as found in the Instructions for Authors”.
