# Supplementary material for: Assessing the off-target movement of tebufenozide in forested ecosystems: implications for vernal pond ecosystems
Source: Environ Monit Assess. 2026 Jan 3;198(1):82. doi: 10.1007/s10661-025-14908-4 (PMC12764619; doi:10.1007/s10661-025-14908-4)

Title: Assessing the off-target movement of tebufenozide in forested ecosystems: Implications for vernal pond ecosystems

Journal Name: Environmental Monitoring and Assessment

Mason S. Ward^1^ (corresponding author), Hlengilizwe Nyoni^2^, Odette Mina^2^, Jon N. Sweetman^1^

^1^Department of Ecosystem Science and Management, The Pennsylvania State University, University Park, Pennsylvania, USA

^2^Energy and Environmental Sustainability Laboratories, Institute for Energy and the Environment, The Pennsylvania State University, University Park, Pennsylvania, USA

E-mail addresses: msw5688@psu.edu; hvn5148@psu.edu; oom5021@psu.edu; jfs6745@psu.edu

ORCID IDs:

Mason S. Ward – 0000-0003-2699-7497

Hlengilizwe Nyoni - 0000-0003-0078-2906

Odette Mina - 0000-0002-0651-9837

Jon N. Sweetman – 0000-0002-9849-7355

**Experimental section**

1. **Materials and standards**

High purity chemicals and solvents, including methanol, water, formic acid, and Pierce ESI positive ion calibration solution were used and were sourced from Fisher Scientific. Tebufenozide Standard was sourced from LGC Dr. Ehrenstorfer (North Charleston, SC 29418, USA)

1. **Sample receipt and handling**

Samples were shipped to the Penn State Environmental Contaminants Analytical Laboratory (ECAL) in cooler boxes filled with ice to prevent degradation of sensitive components. Upon receipt of the samples and chain-of-custody documents, samples were examined, logged-in and then stored in a refrigerator maintained at 4°C. Samples were extracted within 14 days of collection, and to maintain analytical consistency and minimize potential changes in extract composition, all samples were analyzed within 48 hours of extraction. Ten Tebufenozide calibration standards were prepared, levels ranging from 0.01 ng/mL to 10 ng/mL and used to calibrate a Thermofisher Scientific Q Exactive LC-MS, the instrument used to measure concentrations of the samples. Tebufenozide calibration curve with (R^2^ = 0.9998) was generated prior to analyzing the samples and measuring concentrations.

1. **Sample preparation**
   1. ***SPE extraction for water samples***

Water samples were extracted using Oasis HLB cartridges (Waters Corp., Milford, MA) using a vacuum manifold (Supelco Inc., Bellefonte, PA) equipped with Tygon tubing to facilitate efficient sample transfer and minimize contamination. Cartridges were sequentially conditioned with 2 x 7.5 mL of methanol and 2 x 9 mL of UHPLC-grade water at a flow rate of 6–9 mL/min (equivalent to 2-3 drops per second). Subsequently, 250 mL water samples were spiked with 10 µL of 200 ng/mL acetamiprid-D3 as the extraction surrogates and manually agitated for approximately 30 seconds. The samples were loaded onto the SPE cartridges via the Tygon line at a controlled flow rate of 5 mL/min. Following sample loading, each sample bottle was rinsed twice with 7.5 mL of reagent grade water, and the rinsates were transferred to the cartridges using the same tubing. A 5-minute vacuum drying step was then applied under high vacuum conditions (10–15 inHg). The SPE manifold was fitted with a collection rack holding 15 mL glass collection tubes, and elution was performed in two stages. In the first stage, 4 mL of methanol was added to each sample bottle to initiate elution. Each bottle was capped and the solvent was thoroughly swirled to ensure complete rinsing of the container walls with methanol. The Tygon sample transfer lines were then disconnected from the SPE cartridges, and the methanol solutions were carefully transferred into the respective cartridges. Elution was carried out under gravitational flow, allowing analytes to pass into the collection tubes. A second rinse was performed using an additional 4 mL aliquot of methanol, which was transferred to the cartridges via the Tygon. With the vacuum engaged and adjusted to a low draw (<1 inHg), eluents were evaporated to dryness under a gentle stream of nitrogen at 55°C. Dried extracts were spiked with 10 µL of a 200 ng/mL thiacloprid-D4 internal standard and reconstituted to a final volume of 1 mL using a 50:50 methanol/water solution. The reconstituted extracts were then transferred to a 2 mL amber glass autosampler vial, capped, and stored at 4°C for subsequent analysis within 28 days of extraction using LC-MS/MS.

- 1. ***Extraction for Sediment Samples***

***QuEChERS***

Sediment samples were extracted using a modified AOAC method 2007.01 QuEChERS protocol. A 10 g aliquot of homogenized sediment was weighed into a 50 mL polypropylene centrifuge tube, and the remaining homogenized portion of the sample was resealed and returned to frozen storage for potential reanalysis. Each sample received 5 mL of reagent-grade water and 15 µL of acetamiprid-D3 surrogate standard (1000 ng/mL). Tubes were recapped, vortexed for 1 minute, and allowed to stand for 15 minutes. Subsequently, two stainless steel grinding balls and 15 mL of 1% acetic acid in acetonitrile (v/v) were added. After recapping, samples were vortexed for an additional minute. The QuEChERS extraction salt pack (4000 mg MgSO₄ and 1000 mg NaCl) was then slowly added, followed by agitation in a Geno grinder at 1500 strokes per minute for 1 minute. Samples were centrifuged at 4500 rpm for 5 minutes, and the formation of three distinct layers was confirmed to verify successful extraction. The resulting supernatants were subjected to a dispersive solid phase extraction (dSPE) cleanup.

***Sample Cleanup steps (dSPE)***

An 8 mL aliquot of the supernatant (upper organic solvent layer) was transferred to the dSPE tube (900 mg MgSO4, 300 mg PSA, and 150 mg GCB), and one stainless steel grinding ball was added. The dSPE tube was immediately recapped and vortexed for 30 seconds. Following centrifugation at 4500 rpm for 5 min, a 5 mL aliquot of the supernatant was transferred to a glass test tube and evaporated to dryness at 55°C under a gentle stream of nitrogen. The dried residue was reconstituted in a 50:50 methanol/water solution and spiked with 10 µL of 200 ng/mL thiacloprid-D4 internal standard solution, yielding a final volume of 0.5 mL.

1. **UHPLC – MS/MS Conditions**

The separation of the extracted tebufenozide was carried out using a Vanquish UHPLC (Dionex Softron GmbH, Dornierstrasse 4, d-82110 Germany) equipped with an Accucore RP-MS, 150 mm x 2.1 mm, 2.6 µ Particle size (Thermo Fisher Scientific Baltics, V. A. Graičiūno g. 8, 02241 Vilnius, Lithuania). Care was taken to use UHPLC-MS grade solvents. Formic Acid from a Nalgene Poly bottle is known to increase background; therefore, 1 mL glass ampules of formic acid Optima LC-MS Grade were used instead. To prepare a 0.1 % formic acid water solution (Mobile phase A), 1 mL of formic acid was added to 999 mL of UHPLC-MS grade water, and the solution was mixed thoroughly. For Mobile phase B, 1 mL of formic acid was added to 999 mL of UHPLC-MS grade acetonitrile, both of which were sonicated for 15 minutes, and then used as the eluent. The autosampler temperature was set at 4°C, and the column temperature was maintained at 40°C. The injected sample volume was 100 µL. The chromatographic method was programmed and optimized as follows: the initial mobile phase composition (2% B) was maintained for 1 minute, followed by a linear gradient from 2% B to 100 % B in 9 minutes, 100% B was maintained for 2 minutes before being dropped back to 2% B within 0.1 mins and kept constant at 2% B for 1.9 mins. The flow rate was set at 0.4 mL/min, with a total run time of 14 mins. The UHPLC system was connected to a high-resolution, accurate mass (HRAM) Q Exactive Orbitrap Mass spectrometer (Thermo Fisher Scientific, Hanna-Kunath-Str. 11, 28199 Bremen, Germany) equipped with heated electrospray ionization (HESI) and was run in positive ion mode. High-resolution, accurate-mass (HRAM) capabilities provided the ruggedness and sensitivity required for MS/MS-based methods, enabling excellent full-scan quantitation of target compounds with MS/MS confirmations. LC-HARM accurate mass spectra were recorded across the range 65–750 m/z. The data recorded was processed with Thermo Scientific TraceFinder™ 5.1 software. The Q Exactive system was calibrated and tuned using a Thermo Scientific PierceTM ESI positive ion calibration solution delivered by a Chemyx Fusion 101 external pump (Chemyx, Inc 10905 Cash Rd, Stafford, TX 77477). The electrospray ionization was set at 3.5 kV (for positive) with an auxiliary gas set at 5 arbitrary units, the sheath gas set at 36 arbitrary units, and the capillary temperature was set at 320°C. The scan parameters for the mass spectrometer included a run time of 13 minutes and a 6-second chromatogram peak width in DDA mode. MS1 used the Orbitrap mass analyzer with a resolution of 70,000, a maximum injection time (MIT) of 300 ms, one scan, an RF lens (%) of 50, and a scan range from 65 to 750 m/z. The Automatic Gain Control (AGC) target was set to 3e6. MS2 data were acquired using a resolution of 17,500, MIT of 80 ms, AGC target of 1e5, and a scan range from 65 to 750 m/z. The top 1 abundant precursor within an isolation window of 1.0 m/z was chosen for MS/MS analysis. A minimum intensity threshold of 1.0e5 and dynamic exclusion of 6s were used during the data-dependent scanning. For precursor fragmentation, high-energy collision dissociation (HCD)-normalized three-step collision energy was set to 10, 30, and 60.

**Table S1** High Performance Liquid Chromatography Acquisition Settings

| **Parameter** | | **Settings** | |
| --- | --- | --- | --- |
| Analytical column | | Accucore RP-MS, 150 mm x 2.1 mm, 2.6 µ Particle size (Thermo Fisher Scientific) | |
| Column Temp (°C) | | 40 | |
| Mobile phase A | | 1 mL (0.1% v/v) formic acid in 999 mL (99% v/v) UHPLC-MS grade water | |
| Mobile phase B | | 1 mL (0.1% v/v) formic acid in 999 mL (99% v/v) UHPLC-MS grade acetonitrile | |
| Flow rate (mL/min) | | 400 (µL/min) | |
| Run time (min) | | 14 mins | |
| Autosampler temperature (°C) | | 4 | |
| Injection volume (μL) | | 100 | |
| **Time (minutes)** | **Mobile phase A (%)** | **Mobile phase B (%)** | **Flow Rate (µL/min)** |
| 0 | 98 | 2 | 400 |
| 1 | 98 | 2 | 400 |
| 10 | 0 | 100 | 400 |
| 12 | 0 | 100 | 400 |
| 12.1 | 98 | 2 | 400 |
| 14 | 98 | 2 | 400 |

**Table S2** High-resolution, Accurate Mass (HRAM) Q Exactive Orbitrap MS Full MS/ data dependent Acquisition Settings

| **Parameter** | **Settings** |
| --- | --- |
| **HESI source** |  |
| Sheath gas flow (Arbitrary value) | 50 |
| Auxiliary gas flow (Arbitrary value) | 13 |
| Auxiliary gas heater temperature (°C) | 425 |
| Sweep gas flow rate (Arbitrary value) | 3 |
| Spray voltage (\|KV\|) | 3.5 |
| Capillary temperature (°C) | 263 |
| S-lens RF level | 50 |
| **Full Scan Analysis** |  |
| Resolution (FWHM) | 70000 |
| AGC Target | 3e6 |
| Maximum ion time (MIT) | 300 ms |
| Mass Scan Range | 65 – 750 m/z |
| Ion polarity | positive |
| **ddMS2 Analysis** |  |
| Resolution (FWHM) | 17500 |
| AGC Target | 1e5 |
| Maximum ion time (MIT) | 80 ms |
| Loop count | 1 |
| Isolation width | 1 m/z |
| (N)CE/stepped CE | 10, 30, 60 |
| **dd Analysis** |  |
| Minimum AGC Target | 8e3 |
| Dynamic exclusion | 6 s |

**Table S3** Method analytes and associated, retention times, MS/MS precursor ions, and product ions used as confirming ions

| **Type of compound** | **Compound** | **Formula** | **Retention time (mins)** | **precursor (m/z)** | **Fragment m/z)** |
| --- | --- | --- | --- | --- | --- |
| **Target** | Tebufenozide | C22H28N2O2 | 7.70 | 353.2224^a^ | 297.1591  133.0647 |
| **Surrogate** | Acetamiprid_D3 | C10D3H9ClN4 | 4.67 | 226.0933^a^ | 169.0757  161.0027  127.0156 |
| **Internal standards** | Thiacloprid_D4 | C10D4H5ClN4S | 5.15 | 257.0560^a^ | 126.0107  128.0077  99.0000  90.0343 |
| **^a^ ion used for quantitation** | | | | | |

**Quality control and quality assurance**

Ultra-trace clean bottles and glassware were baked in a muffle furnace at 480 °C for 8 hours prior to use in method blanks and laboratory control samples, ensuring the absence of residual tebufenozide contamination. Ongoing precision and recovery standards (OPRs) were prepared and analyzed identically to field samples. Each OPR consisted of 250 mL of UHPLC-MS grade water (Milli-Q IQ 7005 system) and 10 g of reagent-grade sand (fired SiO2 from Alfa Aesar, Ward Hill, MA 01835, USA), spiked with tebufenozide at concentrations of 20 ng/mL and 100 ng/mL. Surrogates were added as follows: 10 µL of 200 ng/mL acetamiprid-D3 for water and 15 µL of 1000 ng/mL acetamiprid-D3 for sand. OPRs were used to evaluate method accuracy and precision. Method blanks were prepared using UHPLC-MS grade water for aqueous samples and fired reagent sand for sediment samples. These blanks were processed identically to field samples, including exposure to laboratory equipment and preservation procedures, to assess potential laboratory-based contamination. No tebufenozide interference was detected in any laboratory blanks. Surrogate recoveries averaged between 75% and 128%, (n=18) and the percent recovery of tebufenozide in water ranged from 77% to 102% (n= 12). In sediments, the percent recovery of tebufenozide ranged from 44% to 74% (n=12). All concentrations were reported without correction for recovery.

**LOD/LOQ**

The limit of detection (LOD) was calculated by multiplying the standard error of the calibration curve intercept by a factor of 3 and dividing the result by the slope. The limit of quantification (LOQ) was calculated similarly, using a factor of 10. Based on these calculations, the LOD was determined to be 0.03689 ng/mL, and the LOQ was 0.1117 ng/mL. For sediment samples (10 g), the LOD and LOQ were converted to 0.37 ng/Kg and1.12 ng/Kg respectively. These values were derived by dividing the original ng/mL concentrations by a preconcentration factor of 10, followed by unit conversion from ng/mL to ng/Kg by first diving by the sample mass (10g), then multiplying the result by 1000). For water samples (250 mL), the LOD and LOQ were calculated as 0.15 ng/L and 0.45 ng/L respectively. These were calculated by dividing the ng/mL values by a preconcentration factor of 250 and converting units to ng/L by multiplying the result by 1000.

Table S4: Physical characteristics of 41 vernal ponds in central Pennsylvania, USA. Variables include water depth (cm), latitude, longitude, spray status as designated by the Pennsylvania Department of Natural Resources, and tebufenozide concentrations in water and sediment. Data are presented for each pond, with “ND” indicating non-detectable concentrations. One water sample contained a value below the limit of quantification (LOQ)


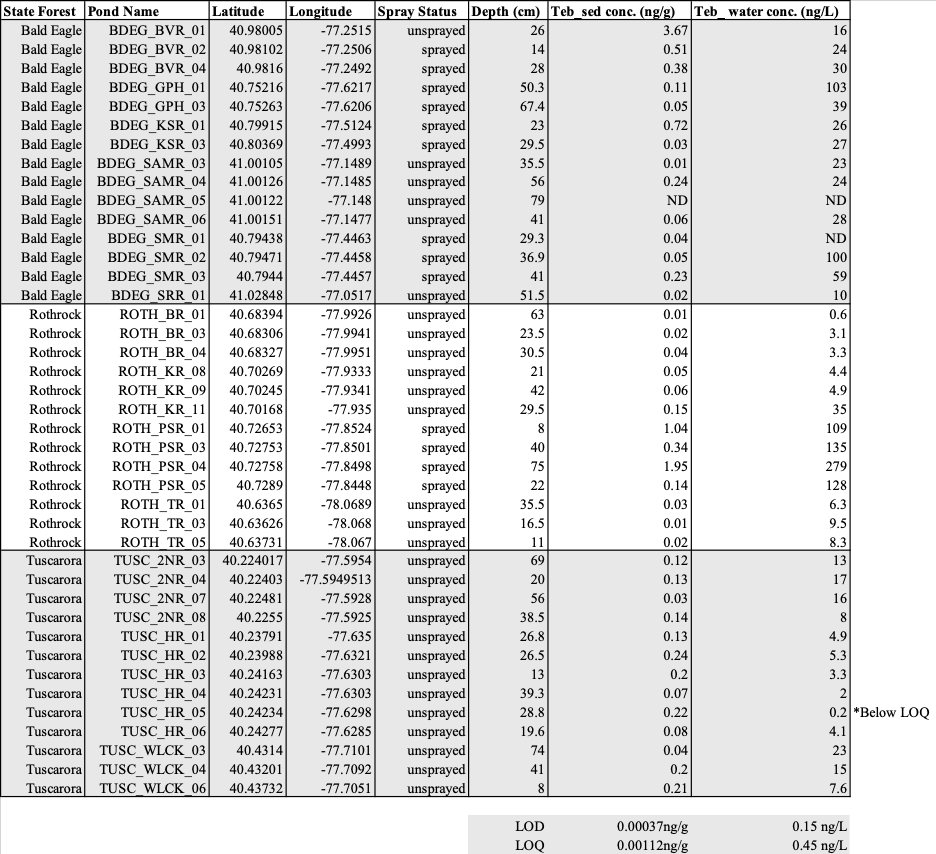

Supplement: Supplementary file 1 — Supplementary Material 1 (DOCX 263 KB) [file 10661_2025_14908_MOESM1_ESM.docx]
